# Research Progress in Imaging Technology for Assessing Quality in Wine Grapes and Seeds

**DOI:** 10.3390/foods11030254

**Published:** 2022-01-18

**Authors:** Francisco J. Rodríguez-Pulido, Ana Belén Mora-Garrido, María Lourdes González-Miret, Francisco J. Heredia

**Affiliations:** Food Colour and Quality Laboratory, Facultad de Farmacia, Universidad de Sevilla, 41012 Sevilla, Spain; rpulido@us.es (F.J.R.-P.); amgarrido@us.es (A.B.M.-G.); heredia@us.es (F.J.H.)

**Keywords:** grape bunches, grape seeds, hyperspectral imaging, chemical imaging, chemometrics

## Abstract

The chemical composition of wine grapes changes qualitatively and quantitatively during the ripening process. In addition to the sugar content, which determines the alcohol content of the wine, it is necessary to consider the phenolic composition of the grape skins and seeds to obtain quality red wines. In this work, some imaging techniques have been used for the comprehensive characterisation of the chemical composition of red grapes (cv. Tempranillo and cv. Syrah) grown in a warm-climate region during two seasons. In addition, and for the first time, mathematical models trained with laboratory images have been extrapolated for using in field images, obtaining interesting results. Determination coefficients of 0.90 for sugars, 0.73 for total phenols, and 0.73 for individual anthocyanins in grape skins have been achieved with a portable hyperspectral camera between 400 and 1000 nm, and 0.83 for total and individual phenols in grape seeds with a desktop hyperspectral camera between 900 and 1700 nm.

## 1. Introduction

### 1.1. Wine Phenolics

The quality of wine depends on the state of different components of the grape at harvest. However, at the same time, these components also vary qualitatively and quantitatively throughout the maturation of the berries. During this maturation, the phenomenon of veraison occurs, which leads to the loss of chlorophyll and the appearance of anthocyanin pigments in the skin. These anthocyanins will give the characteristic colour of red wine. Meanwhile, both in the skin and seeds, other uncoloured phenols are synthesised and develop through polymerisation reactions that modulate the astringency of tannins in the berries and improve the taste quality of grapes. These uncoloured polyphenols also play an essential role in the stabilisation of the colour of red wine, as they interact with anthocyanins and protect their structure through the phenomenon known as co-pigmentation [1]. This interaction is a non-covalent bond resulting in the formation of a charge-transfer complex, π–π, which occurs when two dissolved substances have aromatic rings with very different electronic densities [2]. Modification of wine colour by this phenomenon produces abnormalities in the Lambert–Beer law and has been thoroughly studied, not only with specific absorbance measurements at fixed wavelengths but also considering the entire visible spectrum and evaluating the colour holistically [3]. All factors that depend on the phenolic maturity of the grapes are influenced by agronomic conditions such as climate, soil, water availability, fertilisation, and variety. [4,5]. In areas with warm climates, such as southern Spain, vine growing and the production of quality wines face problems related to high temperatures. In dry springs and hot summers, the gap between the synthesis of sugars in the pulp and the maturity of the solid parts increases. This decoupling makes it difficult to reach the correct aromatic and phenolic maturity in grapes, which eventually leads to poor and irregular colours [6]. Chemical analyses are the most widely accepted reference methods for evaluating seeds. However, these methods are frequently destructive, require lengthy preparation procedures, and involve a large number of samples. Sensory analysis is another approach to assess the condition of the seeds. However, this technique is time consuming and difficult to perform objectively [7]. Optical techniques solve all these disadvantages, offer a rapid and accurate way to evaluate products in the wine industry, and allow winemakers to make quick decisions on harvesting or ageing.

### 1.2. Spectroscopic Techniques for Inspection in the Wine Industry

The use of molecular absorption spectrophotometry is the simplest way to use spectroscopic techniques in the wine industry. For example, a direct measurement of absorbance at 280 nm can estimate the total phenol content, or a measurement at 360 nm can help to analyse the flavonol content. Total pigments can be measured at 520 nm in the visible region of the spectrum. However, a very large number of compounds may be present sharing the same chromophore, so more advanced analytical techniques such as high-performance liquid chromatography (HPLC) are necessary, which again have the disadvantages of chemical analyses. Advances in both instrumentation and computing power to apply chemometrics to large amounts of data have led to the development of vibrational spectroscopic techniques for the analysis of wine as well as different parts of the wine grape.

In wine, there are two main absorption bands at 1450 and 1950 nm related to the molecule of the vibrational response of the O–H bond present in water and ethanol. However, these bands do not offer significant information on phenolic composition. All phenolic compounds that have multiple bonds in common have a simultaneous impact on a small region of the spectrum. By selecting the suitable wavelengths in multivariate chemometric techniques, it was possible to predict some families of compounds in wine [8]. Other studies using infrared spectroscopy could analyse wines from different varieties and having different times of skin-contact maceration [9]. In fact, it can also be used to monitor the winemaking process [10].

Regarding grapes, a vis–NIR spectrophotometer has been used for quantifying condensed tannins in homogenates [11] and for identifying the variety and the origin [12]. In another study, the authors used multivariate regressions to predict flavanols, flavonols, phenolic acids, anthocyanins, and total phenolics on Graciano grape skins [13]. Using ATR-FTIR, Nogales-Bueno et al. analysed the extraction ability of phenolic compounds in grape skins [14].

Grape seeds, as an essential source of flavanols, have also been studied by spectroscopic techniques [15]. As in other cases where indirect analytical techniques are developed, reference analyses are necessary to build the prediction models and to evaluate the goodness of these models. In this case, HPLC was used as a reference method.

NMR spectroscopy has also been successfully used in the wine industry. This way, the biochemical changes during grape ripening [16], the vintage effects [17] or geographical features in wine [18], or even the metabolite fingerprinting in wine-derived products such as vinegar or pomace [19] could be studied.

### 1.3. Imaging Techniques

The wine industry was also revolutionised when imaging techniques arose. Making a machine capable of seeing has always been one of the great challenges of electronics. A camera can record a scene, but it can also understand what it is seeing. Depending on the kind of information they receive, we can differentiate between RGB cameras and hyperspectral cameras [20].

#### 1.3.1. Standard RGB Cameras

The preliminary studies focused on the colorimetric effects of grape and seed maturation and, as common in the agri-food industry, CIELAB colour space was used for this purpose. The chromatic characteristics, the heterogeneity of the colour, and the morphological parameters were useful for discriminating the variety and the kind of soil of these cultivars and for monitoring the maturation process and establishing critical points along this maturation [21]. Another study, using the same method, could discriminate grape seeds when an early leaf removal was applied to the vineyard [22]. When the chemical composition was added to the colorimetric information, it was possible to predict the concentration of hydroxybenzoic acids, flavanol monomers, procyanidin dimers, procyanidin trimers, and galloylated flavanols [23].

Artificial intelligence has also changed the way of evaluating vines with RGB cameras. From images of bunches acquired with the camera of a smartphone and with a homogeneous black background, it has been possible to estimate the number of berries in bunches of grapes successfully [24]. Through neural networks and support vector machines, they were able to make reliable models for multiple varieties of white and red grapes. The methodology developed by the authors has been implemented in the vitisBerry app [25], which works on Android devices. This algorithm has been improved by the same authors, who have recently created a system consisting of a camera mounted on an off-road vehicle that acquires images directly in the vineyard [26]. Images obtained at night were easier to segment, since there was no parasitic radiation and only the nearest objects were illuminated. When estimating the yield of the vines by this system, a correlation coefficient of 0.98 was obtained when comparing the number of predicted berries with the actual value. In another study and with special cameras, it was possible to perform a 3D reconstruction of the morphology of the bunches (enabling the discernment of the internal stems) and to study their phenotype [27].

#### 1.3.2. Hyperspectral Cameras

Imaging techniques have successfully adapted spectroscopy with the rise of hyperspectral imaging, making it possible to evaluate both the chemical composition and its distribution on the surface of a sample [28]. The concept of hyperspectral imaging is far removed from everyday life, but its applications are very beneficial in the fields where it has been applied, such as agriculture, medicine, mineralogy, astronomy, or fine arts. To improve its comprehension, it can be compared with a conventional colour image. A common image has three bands, red, green, and blue, and the combination of these produces the rest of the colours. Similarly, a hyperspectral image has a high number of bands, each corresponding to a unique region of the electromagnetic spectrum. These bands do not have to belong to the visible region, so a hyperspectral image can also include the near infrared, where the relationship between spectral characteristics and chemical composition is even closer. Thus, the information obtained with these cameras goes beyond what a conventional digital camera can achieve. This way, we could define a hyperspectral image or hypercube as a stack of images of the same scene, where each represents, in greyscale, the reflectance at a single wavelength. Each pixel of the image contains a full spectrum of that position, which is stored as a fingerprint. This arrangement makes the hypercube have three-dimensional information, where two of the dimensions correspond to spatial coordinates (x,y) and the third dimension corresponds to the spectral dimension (λ), which will be related to the chemical composition [29,30].

Hyperspectral images have also been used successfully for the characterisation of solid parts in the wine industry. In an initial study, it was possible to differentiate between red and white grape seeds, different red grape varieties, and even agronomic factors such as soil type within the same white grape variety could be distinguished [31]. It has also been possible to study the concentration of flavanols in grape seeds and their extractability using the same technology [32]. Another study evaluated the NIR spectrum of more than a thousand seeds individually, grouped them according to their spectral similarity, and analysed their flavanolic profile, demonstrating that seed maturation does not occur at the same time but that seeds evolve to a different degree, causing a certain chemical heterogeneity at each moment of maturation [33]. The technique has also been effective for the characterisation of red grapes, where, in addition to the anthocyanin profile of the skin [34], other characteristics related to the technological maturity of the pulp were analysed [35,36]. Regarding the obtention of chemical images, Agati et al. used a CCD camera equipped with a motorised filter wheel to obtain chlorophyll fluorescence images from whole grape bunches [37].

Whenever hyperspectral cameras were used to evaluate chemical composition, the studies were carried out in the laboratory and under controlled lighting [38]. To our knowledge, there are no studies in which hyperspectral technology has been used in the field for chemical analysis. Therefore, the main objective of this article has been to improve the chemical analysis of the different parts of the grape using imaging techniques and, on the other hand, to extrapolate the multivariate statistical models developed in the laboratory to images acquired in the vineyards.

## 2. Materials and Methods

### 2.1. Sampling

Grapes (*Vitis vinifera* L.) from the two vineyards sampled are included under the “Condado de Huelva” Designation of Origin, in Southwestern Spain, harvested in 2020 and 2021. The fields in Condado de Huelva are flat or weakly undulated, and their medium fertility and neutral or lightly basic characteristics make them an ideal place to grow grapes. The first vineyard belonged to the Tempranillo variety (37°19′09″ N 6°35′56″ W) and another belonged to Syrah (37°17′51″ N 6°36′59″ W). These were taken twice a week from early July to harvest (which occurred approximately mid-August depending on the variety and the state of maturation).

Sampling consisted of the acquisition of hyperspectral images of whole bunches throughout the vineyards. In two of these bunches, the sugar content of five different grapes per bunch was measured and labelled on the images to ensure traceability of the results. Furthermore, from each vineyard, two other bunches were carefully cut from the stem and transferred to the laboratory under refrigeration. Once in the laboratory, images were acquired again, and skins and seeds were separated and stored frozen until chemical analyses were performed.

### 2.2. Images Acquisition

#### 2.2.1. DigiEye Imaging System

This apparatus includes an illumination box specially designed to illuminate the samples consistently to measure colour and a digital camera connected to a computer [39]. The cabinet is equipped with two fluorescent tubes that emulate the standard illuminant D65 and offer stable lighting conditions. Lamps were switched on at least 10 minutes before being used, according to manufacturer indications, to stabilise them. This equipment was used in the laboratory to acquire images of entire bunches of grapes and their manually separated seeds.

#### 2.2.2. Hyperspectral Measurements

NIR hyperspectral images were acquired with equipment that comprise the following parts: Xenics^®^ XEVA-USB InGaAs camera (Xenics Infrared Solutions, Inc., Leuven, Belgium); Specim ImSpector N17E Enhanced spectrograph, which covers the range between 884 and 1717 nm (Spectral Imaging Ltd., Oulu, Finland); and a computer system to run SpectralDAQ 3.62 acquisition software (Spectral Imaging Ltd., Oulu, Finland). As illumination, two 70 W tungsten iodine halogen lamps at 45° were used. A “white reference” image (W, 100% reflectance) was acquired from a white ceramic tile (Labsphere Inc., North Sutton, NH, USA), and a “dark reference” image (B, 0% reflectance) was obtained with the light source off and the camera covered with its opaque cap. The white and dark “reference” images were used to correct the raw images (R_0_) to obtain a relative reflectance image (R) according to the equation R = (R_0_ − B)/(W − B). Further description, as well as a scheme of this equipment, is available in Nogales-Bueno et al. [40]. This camera was used in the laboratory to acquire images of entire bunches of grapes and their extracted seeds.

The Specim IQ hyperspectral camera (Spectral Imaging Ltd., Oulu, Finland) is also a linear camera based on the push-broom principle that includes in one body all parts necessary to acquire images with a resolution of 512 × 512 pixels and 204 bands in the range 400–1000 nm. The main advantage is that it works with rechargeable batteries and can be used as a portable device. For the images acquired in the laboratory, the same lamps as those used for the NIR camera were used. In the case of the field images, the images were acquired in direct sunlight during the early hours of the morning. This camera has a certified reflectance device to perform the calibrations, so different white measurements were made throughout the sessions in order to correct daylight changes. In all cases, the integration time was adjusted to obtain a good spectral sensitivity in all the images. A very detailed description can be found in Behmann et al. [41]. This camera was used in both the laboratory and the field to acquire images of entire bunches of grapes.

### 2.3. Chemical Analyses

#### 2.3.1. Sugar Content in Must

The concentration of sugars in grape must is usually measured by densitometry, as the density changes according to its concentration. In our study, we needed to measure the sugar content of individual grapes, so it was impossible to use this technique. Therefore, the sugar concentration was measured with a portable Abbe refractometer from some drops of must extracted manually from individual grapes [42]. This analysis led to a one-to-one correspondence between the grape spectrum and its technological maturity.

#### 2.3.2. Extraction and Analysis of Phenolics in Grape Seeds and Skins

For these chemical analyses, it was necessary to perform a previous extraction of grape seeds and grape skins in wine-like medium (12% (*v*/*v*) ethanol, 5 g/L tartaric acid, 11.48 g/L sodium chloride, and pH 3.6). The extractions were performed on the model wine so that the results would be comparable with the actual extractable content of seeds and skin in the wine during the winemaking process. A ratio of 25 mL of model wine per gram of seed was used. Skin extraction was carried out in a 20 mL proportion of model wine for each gram of skin. These macerations were carried out at room temperature for 72 h and without external agitation. These extractions were performed in triplicate, and the supernatants were analysed to determine the extractable content.

The extractable total phenolic content was determined using the Folin–Ciocalteu method [43]. Briefly, 100 µL of model wine supernatants were mixed with 1.5 mL of sodium carbonate (20% *w*/*v*) and 500 µL of Folin reagent (Panreac Química S.L.U., Barcelona, Spain) and made up to the volume with deionised water in a 10 mL volumetric flask. After 2 h of incubation at room temperature, the solutions were measured at 765 nm using an Agilent UV–vis HP8453 spectrophotometer (Palo Alto, CA, USA). Gallic acid was used as the standard, and the results were expressed as gallic acid equivalents (mg GAE/g of grape seed or skin).

To measure the extractable total flavanol content, a modification of the method of Vivas et al. [44] was applied. The DMACA (4-dimethylaminocinnamaldehyde) reagent (Sigma-Aldrich, Madrid, Spain) was prepared immediately before use, containing 0.1% (*w*/*v*) DMACA in a mixture of hydrochloric acid and methanol (1:10 *v*/*v*, respectively). Subsequently, 10 or 20 µL (depending on the concentration) of model wine supernatants were mixed with 190 or 180 µL of methanol, respectively, and 1 mL of DMACA reagent. The absorbance of the mixtures was measured at 765 nm on an Agilent 8453 UV–visible spectrophotometer (Palo Alto, CA, USA), using a calibration curve of (+)-catechin for its quantification. The results were expressed as mg of catechin equivalents per gram of grape seed or skin.

In both the Folin–Ciocalteu and DMACA analyses, dilutions were carried out when the concentration was outside the linear range of the calibration curve.

HPLC separation, identification, and quantification of phenolic compounds from model wine supernatants were carried out on an Agilent 1200 chromatographic system equipped with a quaternary pump, a UV–vis diode array detector, an automatic injector, and ChemStation software (Palo Alto, CA, USA). Samples were filtered through 0.45 µm pore size filters and injected directly into the chromatographic system.

Individual extractable phenolic compounds (flavan-3-ols, flavonols, hydroxycinnamic acid derivatives, and other low-molecular-weight phenolic compounds) were identified in model wine supernatants from grape seeds and grape skins by a modification of the method described by Castillo-Muñoz et al. [45]. These were separated by injecting a volume of 50 µL, of each sample, into a Zorbax C18 column (250 × 4.6 mm, 5 µm particle size) at 40 °C. Solvent A consisting of acetonitrile–formic acid–water (3:10:87) and solvent B of acetonitrile–formic acid–water (50:10:40) were used. The elution profile was as follows: 0–5 min 94%A–6%B; 5–10 min 89%A–11%B; 10–15 min 89%A–11%B; 15–20 min 80%A–20%B; 20–25 min 77%A–23%B; 25–30 min 74%A–26%B; 30–35 min 60%A–40%B; 35–38 min 50%A–50%B; 38–46 min 40%A–60%B; and 46 min 94%A–6%B. The flow rate was 0.63 mL/min. UV–vis spectra were recorded from 200 to 800 nm with a bandwidth of 2.0 nm. Quantification was performed at 280 nm (flavanols and benzoic acids), 320 nm (phenolic acids), and 360 nm (flavonols) by comparing the areas and retention times with the standards of gallic acid, (+)-catechin, caffeic acid, and quercetin, respectively (Sigma-Aldrich, Madrid, Spain). The concentration of extractable phenolic compounds was expressed as mg/g of grape seed or skin.

The identification of extractable anthocyanins in model wine supernatants from grape skins was carried out following the method proposed by Heredia et al. [46]. The injecting volume, the column, and the solvents employed were the same as those used for the identification of individual phenols. The working temperature was 38 °C, and the elution profile was as follows: 0–10 min 94%A–6%B; 10–15 min 70%A–30%B; 15–25 min 60%A–40%B; 25–35 min 55%A–45%B; 35–40 min 50%A–50%B; 40–42 min 40%A–60%B; and 42–43 min 94%A–6%B. The flow rate was 0.8 mL/min. UV–vis spectra were recorded from 200 to 800 nm with a bandwidth of 2.0 nm. Quantification was carried out at 525 nm by comparing the areas and retention times with the malvidin 3-glucoside standard (Extrasynthese, Genay, France). The concentration of extractable anthocyanins was expressed as mg/g of grape skin.

### 2.4. Image Processing and Statistics

All image processing, including segmentation, data extraction, data processing, construction of prediction models, evaluation of these models, as well as the generation of the chemical imaging images, were done with MATLAB R2020a [47].

## 3. Results

### 3.1. Chemical Analyses

The sugar content of 151 individual Tempranillo grapes was analysed and ranged between 8.5 and 21.4 °Brix. For Syrah grapes, the sugar of 182 individual grapes ranged between 4.2 and 20.7 °Brix (data not shown). During the 2020 vintage, Syrah grapes increased their sugar concentration at a constant rate until the end of ripening. On the other hand, Tempranillo grapes increased rapidly in sugar concentration in the early stages, decreasing this rate three weeks before harvest. During the last week and a half of ripening, the grapes of the two varieties did not show statistically significant differences (*p* < 0.05) in sugar concentration. Although the dates did not coincide exactly, the general behaviour of the grapes of the two varieties was similar in the 2021 vintage.

Table 1, Table 2 and Table 3 show the main statistical descriptors for the reference parameters of the grape seeds and grape skin samples. Note that the high standard deviations obtained were not due to measurement errors but to the evolution of the samples during maturation.

On the one hand, in the grape seeds of the Syrah variety, a clear decrease in extractable phenolic and flavanolic compounds (total or individual) was observed throughout the maturation process. On the other hand, the seed samples of the Tempranillo variety did not show a continuous decrease or increase trend in their extractable phenolic and flavanolic compounds during the maturity period. In this case, the concentrations of the extractable compounds studied were more variable throughout this process. Regardless of the variety, the concentration of total phenolic compounds extracted in the model wine ranged from 0.45 to 23.18 mg/g of grape seed, and the concentration of total flavanols ranged between 0.01 and 6.61 mg/g of grape seed (Table 1). These values are similar to those reported in other research articles [32,48].

Among the phenolic compounds extractable in model wine from seeds, several types of compounds have been identified, such as benzoic acids (gallic acid) or flavan-3-ols ((+)-catechin and (−)-epicatechin). Table 1 shows the concentration ranges in which these phenolic compounds were obtained. These chemical compounds extracted in model wine are normally present in wine and are well known [49]. When the two varieties were compared, a higher concentration of total or individual extractable flavanols was obtained in Syrah than in Tempranillo. In the rest of the parameters, both varieties were similar. Table 2 and Table 3 show the data obtained in the skin extraction in model wine.

Regardless of the variety, the concentration of total phenolic compounds, extracted in model wine, ranged from 4.62 to 20.73 mg/g of grape skin, and the concentration of total flavanols ranged between 0.53 and 2.87 mg/g of grape skin. These values were similar to those obtained in other studies [50]. Among the extractable phenolic compounds identified, benzoic acid, flavan-3-ols, hydroxycinnamic acids and their derivatives, and flavonols were found. These compounds are commonly found in wine, as shown in previous research [45,51]. The five expected native grape anthocyanins (delphinidin-3-glucoside, cyanidin-3-glucoside, petunidin-3-glucoside, peonidin-3-glucoside, and malvidin-3-glucoside) and their acetylated and *p*-coumaroyl derivatives were detected in grape skin extraction supernatants [52,53]. These monomeric anthocyanins were extracted at concentrations ranging from 0.00 to 1.86 mg/g of grape skin, with malvidin-3-glucoside being the majority and cyanidin-3-glucoside the minority. These anthocyanins correspond to the majority and minority anthocyanins present in wine as reported by other studies [54].

Neither of the two varieties showed a clear behaviour of the extractable chemical compounds studied throughout the maturation process. However, significant differences were observed between Syrah and Tempranillo. The Syrah variety showed higher concentrations of some phenolic compounds such as quercetin-3-glucoside, isorhamnetin-3-glucoside, and syringetin-3-glucoside. The same was true for some anthocyanins, such as peonidin-3-glucoside and the acetylated and *p*-coumaroyl derivatives of peonidin, petunidin, and malvidin. However, the Tempranillo variety showed higher concentrations for total flavanols, in some anthocyanins (delphinidin-3-glucoside, cyanidin-3-glucoside, and petunidin-3-glucoside) and in some other phenolic compounds such as gallic acid, catechin, t-caftaric acid, t-coutaric acid, and c-coutaric acid. For the rest of the extractable compounds studied, no differences were found between the varieties.

### 3.2. Image Processing

Only in the NIR, a hyperspectral desktop camera was necessary to carry out an image calibration as image preprocessing. The portable hyperspectral camera automatically performed the calibration before each session using a certified reflectance device. For the images acquired in the DigiEye equipment, a calibration was also performed in each session using a certified colour chart, thus obtaining the CIELAB coordinates directly in each pixel. Those hyperspectral images obtained in the laboratory had a homogeneous white surface made of polyethylene as a background, and it was easy to identify the samples in them. However, it was a computational challenge to automatically identify the grapes in the hyperspectral field images. In these images, there were branches, leaves, soil, sky, and other elements present. In addition, the solar illumination caused many shadows, which made this segmentation process difficult. Simple segmentation by thresholding was not possible, so different machine learning methods were tested for this purpose. Reflectance spectra of various elements and multiple images were manually collected and labelled according to the region of membership. Once the models were trained, no satisfactory results were obtained. After several tests, the best performing model was a forward-stepwise linear discriminant analysis that simply split pixels into grape and not grape. This model sequentially introduced nine spectral bands until the inclusion of a new band did not improve the model. The bands in decreasing order of relevance were 409, 478, 560, 675, 694, 760, 902, and 982 nm. This segmentation criterion was used for the portable camera in both laboratory and field images. For NIR hyperspectral images, and since the background had a very characteristic spectral profile, the segmentation was performed by thresholding after subtracting the bands belonging to 1544 and 1517 nm. Finally, the images with colorimetric information (DigiEye) were segmented by thresholding after dividing Chroma (C*_ab_) by Lightness (L*).

Once the images were segmented, the next step was to extract the information of interest from the images. In the case of the seeds, as they were analysed as a group and not individually, an algorithm was programmed to obtain the spectrum and average colour and spectrum of each seed and store it in a table. For the grapes, as in the case of sugars, the sampling unit was an individual grape, and an algorithm was programmed to present each of the images and, where it was necessary, to manually select those grapes sampled. This was the only way to establish a direct correspondence between the grapes and their chemical composition. Furthermore, the mathematical prediction model for entire grapes would not be object based, but pixel based. For this reason, and to improve the capacity of the model, when selecting the centre of the grape for data extraction, it was obtained the spectrum of 21 pixels that made up a target shape matrix [20]. For the seed images, as they were arranged on the sample tray in an ordered way, an object-based strategy was followed, so the training of the models and the obtaining of the results were specific to each seed, not to each pixel.

### 3.3. Prediction Models

The first step in the development of mathematical models was to merge chemical and spectral/colorimetric data. The itemised sample coding allowed this merging to be done automatically. In the case of grapes, a global data matrix was obtained with 4969 rows belonging to individual pixels and, in columns, the following information: sample code, replicate of chemical analysis, object number (grape), pixel number, type of image (laboratory or field), sampling date, chemical composition, colorimetric variables, vis–NIR spectrum (400–1000 nm), and NIR spectrum (900–1700 nm). In the case of seeds, an analogous table with 2462 rows was obtained, each belonging to the average spectrum/colour of one seed. Seeds from each sample had the same chemical result assigned. It had the following information: sample code, replicate of chemical analysis, object number (seed), sampling date, chemical composition, colorimetric variables, and NIR spectrum (900–1700 nm).

To improve the significance of the models and to avoid overfitting phenomena, the samples were divided into calibration and validation sets. For seeds, the samples were distributed 75% for calibration and 25% for validation. For grapes, the distribution was the same. However, in the case of the models built with the portable hyperspectral camera, and as it was one of the objectives of this work, the images obtained in the laboratory were assigned as the prediction set and the images acquired in the field as the validation set.

**Figure 1:**
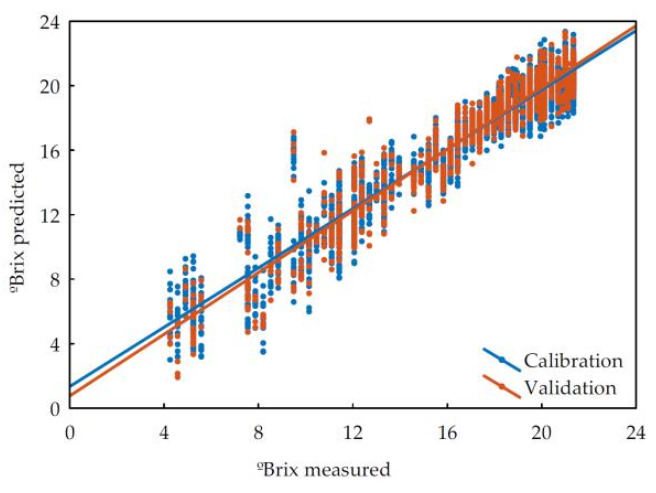
Scatterplot of observed vs. predicted values of °Brix in grapes.

In order to predict the chemical information from imaging data, a partial least squares regression (PLSR) was performed. As many regressions were performed as possible between chemical results and image data. In fact, colorimetric and spectral data were tested together and separately as independent variables to assess whether different imaging data could interact synergistically for predictive purposes. For example, in the case of °Brix prediction and when colorimetric data were used as independent variables, the R^2^_val_ value was 0.74. In the case of using the hyperspectral data from the NIR camera (900–1700 nm), it reached only 0.69 and dropped to 0.58 when converting reflectance data to absorbance. It is worth mentioning that in the case of the DigiEye and hyperspectral NIR cameras, there were no images obtained in the field, only in the laboratory, so their use would have limited the main objective of this work. The data obtained from the portable hyperspectral camera (400–1000 nm) led to the best performance. The R^2^_val_ was 0.88 with the reflectance data and reached 0.91 when converted to absorbance (Figure 1). Spectroscopic pretreatments such as smoothing, standard normal variate, derivatives, and combinations of them were used to improve the performance, but they did not enhance them substantially. In fact, most of them decreased the quality of predictions. The behaviour with the rest of the compounds was similar. The results of the prediction models for the most significant compounds obtained from absorbance spectra with the Specim IQ camera are shown in Table 4.

During the construction of PLRS prediction models, it is assumed that there are no errors in the reference values. However, any analytical determination involves an associated error, which will become larger as the number of steps performed in the analysis increases. Since different analytical methods were used for analysing analytes in different concentrations, the goodness of fit was unequal according to matrix and analyte. Regarding to grape parts, the best prediction was based on must sugar, then seeds, and, finally, skins. Must sugar was the best prediction probably due to the sampling unit of the reference analyses. Brix degree was measured on individual grapes, while the compounds in seeds and skins were analysed in groups according to sampling. This better correspondence between samples and spectral information may have influenced this improvement of the models. Another aspect that may have influenced the quality of the prediction models is the compound concentration. Total phenols were present in the skins with an average concentration of higher than 10 mg/g while total flavanols were present at a lower order of magnitude (~1 mg/g). This difference would affect the reproducibility of the measurements and is reflected in the coefficients of determination (0.73 and 0.51, respectively). Similar phenomena occurred in the seeds. Among the analytes analysed by spectrophotometric techniques, total phenols were more accurately predicted than total flavanols. Similarly, within the analytes determined by HPLC, compounds such as gallic acid and catechin were found in higher concentrations than epicatechin, and this is also reflected in the quality of the prediction models.

Considering families of compounds, total phenol content was predicted with better accuracy than total flavonols. It is noteworthy that compounds such as gallic acid or some anthocyanin monomers could not be measured accurately. In any case, the prediction error was at least one order of magnitude lower than the maximum concentration value obtained in the chemical analysis. The data obtained with the DigiEye equipment did not yield good results. In the case of grapes, the colour change is due to anthocyanin biosynthesis. Once the grapes have changed colour and their appearance is almost black, these anthocyanins continue to evolve, but the colour is too dark to observe changes after this point. In previous works, prediction parameters similar to those of this work were obtained (R^2^_val_ = 0.94 for °Brix) [38]. However, in the cited literature, individual compounds were not predicted, but general parameters were measured in oenological analyses. Moreover, in the cited study, all images were obtained under controlled illumination in laboratory conditions, so these models could not be extrapolated to uncontrolled conditions such as vineyards.

The prediction models applied to seeds performed better than those for whole grapes in bunches, especially for total phenols and catechin, where coefficients of determination between predicted and measured values in the validation samples were greater than 0.8 (Table 4). These prediction models were probably better because all images were acquired in the laboratory and under controlled lighting. Moreover, in these seed samples, the distribution between the calibration and prediction samples was randomised. It is remarkable that, for total flavanols, there was a large difference between calibration and validation data, probably due to some overfitting phenomenon in the model. As with the grapes, the colorimetric data could not be used for the prediction of chemical composition nor did the models improve when the colorimetric variables were added to the spectral data.

Although the relationship between the seed colour and phenolic composition has been demonstrated in previous work [23], the seeds used in this work belong to a warm-climate region, they were not at optimal phenolic maturity when harvested, and the colour had not changed sufficiently. Therefore, there was no optical variability in the samples to make these data useful.

### 3.4. Chemical Imaging

**Figure 2:**
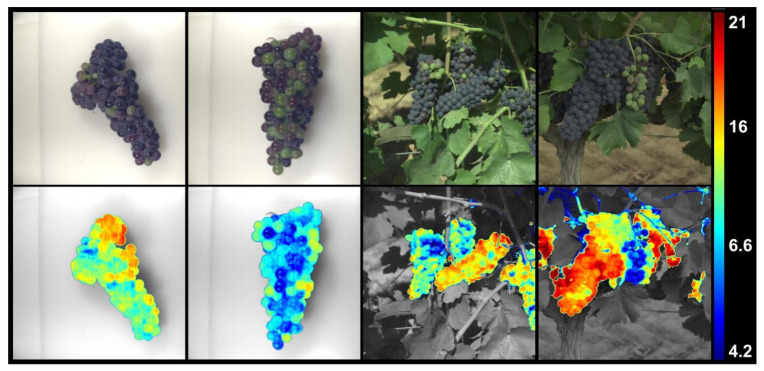
Prediction of °Brix in bunches in laboratory and field hyperspectral images.

Finally, an algorithm was also developed to obtain chemical imaging representations. These images would show, on the one hand, the real appearance of the scene in an RGB image and, in parallel, a greyscale image where, using a pseudo-colour scale, the concentration of any of the analytes for which the models were built would be shown in the segmented area. Each of these images was accompanied by a legend showing the correspondence between concentration and the pseudocolour scale. Figure 2 shows examples of images showing the concentration of sugars expressed as °Brix. As expected, there were differences in sugar content depending on whether the grapes were before or after veraison. However, there were also differences in sugars in grapes with the same appearance. Therefore, a conventional camera could not have been used for this prediction.

**Figure 3:**
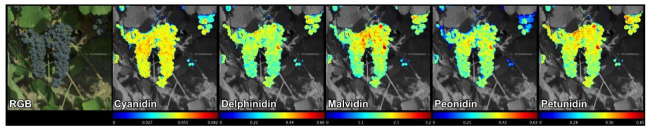
Prediction of anthocyanins-3-glucoside in bunches of a sample of Syrah.

Similarly, the mathematical models for the prediction of the remaining compounds were applied, and chemical images were produced. Figure 3 shows an RGB reconstruction of a hyperspectral image and the predicted concentration of anthocyanin monomers for this sample in each pixel of the image. Each image has a different scale, so concentrations cannot be compared solely on the appearance of each image. However, it is interesting how the distribution of compounds is different across the bunch. Within each bunch, the concentration distribution of cyanidin and delphinidin was more homogeneous, although, in the case of delphinidin, isolated grapes were found to have higher concentrations. For the rest of the compounds, especially malvidin and petunidin, the concentration in the upper part of the bunch was higher than in the lower part. It should be noted that the chemical analysis of the skin of each bunch was done together, not discriminating between the position of the grapes along the bunch. Therefore, it is possible that the goodness of fit would have been improved by decreasing the size of the sample unit.

**Figure 4:**
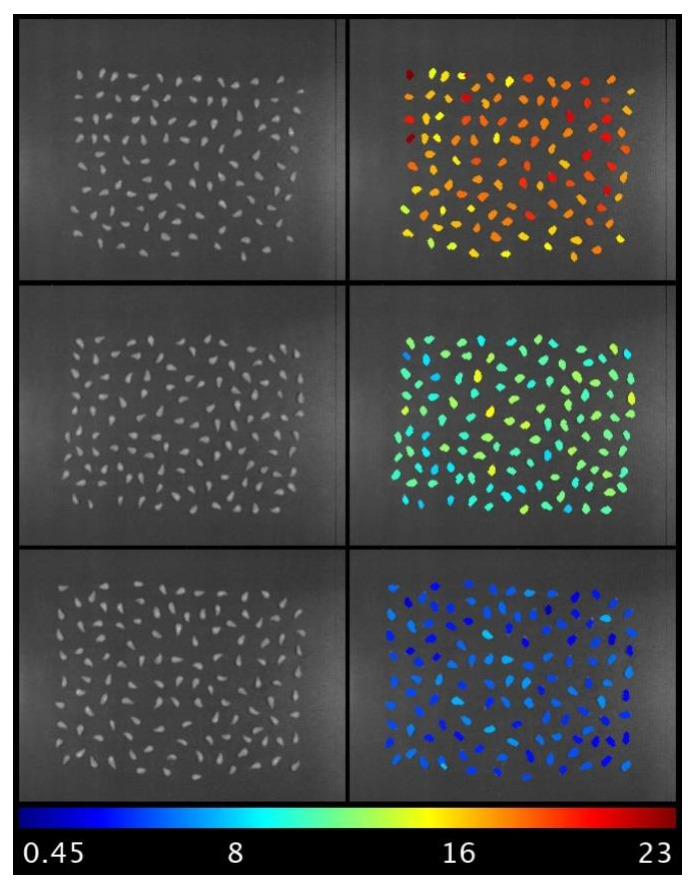
Prediction of total phenols in seeds of Syrah at different moments along maturation.

An algorithm was also developed to obtain chemical images of seeds. Figure 4 shows some examples where, on the left, an infrared band is shown where these seeds are well differentiated from the background and, on the right, the result of this prediction. As demonstrated by Quijada-Morín et al. [33], there is some chemical heterogeneity in grape seeds within the same sample. As ripening progresses, a decrease in the phenolic content of the seeds can be observed.

## 4. Conclusions

In this work, progress has been made in the use of different imaging techniques for the evaluation of wine grapes and wine grape seeds. Among them, hyperspectral techniques proved to be much more useful than conventional image analysis techniques with an RGB camera for this purpose. In the case of whole grapes, it was possible to measure very accurately the sugar content and to estimate families and some individual phenolic compounds from their spectra between 400 and 1000 nm. Furthermore, and for the first time, it has been possible to transfer mathematical models trained on laboratory samples to field samples. In the case of grape seeds, even more reliable mathematical models for the prediction of phenolic compounds were obtained from hyperspectral images in the range between 900 and 1700 nm.

The most significant advance in this work is the possibility of performing quantitative chemical analysis on grape bunches in the vineyard without the need of sampling the bunches. To make the prediction models robust, the images were acquired regardless of environmental conditions such as the amount of sunlight or the presence of shadows produced by the different parts of the vines. These random circumstances may have influenced the goodness of the models. Therefore, further studies should continue by controlling these variables or by applying other non-linear prediction methods or artificial intelligence.

## Figures and Tables

**Table 1:** Summary of chemical analysis of grape seeds (mg/g of grape seed).

Compound	N	Mean	Minimum	Maximum	SD
**Syrah**					
Total phenolic content	57	3.76	0.79	23.18	5.4
Total flavanol content	57	1.23	0.18	6.61	1.73
Benzoic acids:					
Gallic acid	57	0.08	0.04	0.25	0.05
Flavan-3-ols:					
(+)-Catechin	57	0.27	0.03	1.42	0.36
(−)-Epicatechin	57	0.37	0.06	1.57	0.40
**Tempranillo**					
Total phenolic content	51	2.43	0.45	6.31	1.68
Total flavanol content	51	0.67	0.01	3.36	0.74
Benzoic acids:					
Gallic acid	51	0.07	0.04	0.11	0.02
Flavan-3-ols:					
(+)-Catechin	51	0.14	0.02	0.91	0.18
(−)-Epicatechin	51	0.1	0.01	0.41	0.10

**Table 2:** Summary of chemical analyses performed for Syrah grape skins (mg/g of grape skin).

Compound	N	Mean	Minimum	Maximum	SD
Total phenolic content	57	11.67	4.62	20.73	4.58
Total flavanol content	57	0.92	0.53	1.46	0.22
Benzoic acids:					
Gallic acid	57	0.29	0.14	0.57	0.10
Flavan-3-ols:					
(+)-Catechin	57	0.03	0	0.09	0.02
Hydroxycinnamic acid derivatives:					
*t*-Caftaric acid	57	0.01	0	0.1	0.01
*p*-Coumaric acid	57	0.01	0	0.02	0.01
Flavonols:					
Myricetin-3-glucuronide	57	0.04	0	0.18	0.04
Myricetin-3-glucoside	57	0.04	0	0.09	0.03
Quercetin-3-glucuronide	57	0.09	0.01	0.27	0.08
Quercetin-3-glucoside	57	0.16	0.01	0.49	0.13
Laricitrin-3-glucoside	57	0.01	0	0.02	0.01
Isorhamnetin-3-glucoside	57	0.07	0	0.23	0.06
Syringetin-3-glucoside	57	0.03	0	0.13	0.03
Monomeric anthocyanins:					
Delphinidin-3-glucoside	57	0.21	0	0.43	0.07
Cyanidin-3-glucoside	57	0.05	0	0.06	0.01
Petunidin-3-glucoside	57	0.34	0	0.77	0.17
Peonidin-3-glucoside	57	0.4	0	0.77	0.17
Malvidin-3-glucoside	57	1.86	0	4.96	1.30
Petunidin-3-acetyl-glucoside	57	0.22	0	0.35	0.07
Peonidin-3-acetyl-glucoside	57	0.31	0	0.46	0.10
Malvidin-3-acetyl-glucoside	57	1.18	0	2.7	0.75
Petunidin-3-*p*-coumaroyl-glucoside	57	0.3	0	0.59	0.17
Peonidin-3-*p*-coumaroyl-glucoside	57	0.26	0	0.35	0.08
Malvidin-3-*p*-coumaroyl-glucoside	57	0.73	0	1.47	0.40

**Table 3:** Summary of chemical analyses performed for Tempranillo grape skins (mg/g of grape skin).

Compound	N	Mean	Minimum	Maximum	SD
Total phenolic content	51	12.84	7.25	20.62	3.39
Total flavanol content	51	1.65	0.64	2.87	0.45
Benzoic acids:					
Gallic acid	51	0.48	0.2	1.08	0.20
Flavan-3-ols:					
(+)-Catechin	51	0.05	0	0.16	0.04
Hydroxycinnamic acid derivatives:					
*t*-Caftaric acid	51	0.04	0	0.13	0.03
*t*-Coutaric acid	51	0.01	0	0.06	0.01
*c*-Coutaric acid	51	0.02	0	0.06	0.01
*p*-Coumaric acid	51	0.01	0	0.03	0.01
Flavonols:					
Myricetin-3-glucuronide	51	0.04	0	0.11	0.03
Myricetin-3-glucoside	51	0.04	0.01	0.07	0.01
Quercetin-3-glucuronide	51	0.09	0.02	0.41	0.07
Quercetin-3-glucoside	51	0.06	0.01	0.24	0.05
Laricitrin-3-glucoside	51	0.01	0	0.03	0.01
Isorhamnetin-3-glucoside	51	0.01	0	0.02	0.01
Syringetin-3-glucoside	51	0.01	0	0.02	0.01
Monomeric anthocyanins:					
Delphinidin-3-glucoside	51	0.33	0.17	0.66	0.10
Cyanidin-3-glucoside	51	0.06	0.04	0.08	0.01
Petunidin-3-glucoside	51	0.47	0.19	0.85	0.15
Peonidin-3-glucoside	51	0.29	0.19	0.43	0.06
Malvidin-3-glucoside	51	1.75	0.54	3.14	0.57
Petunidin-3-acetyl-glucoside	51	0.19	0.16	0.23	0.01
Peonidin-3-acetyl-glucoside	51	0.17	0.16	0.18	0.00
Malvidin-3-acetyl-glucoside	51	0.35	0.18	0.46	0.07
Petunidin-3-*p*-coumaroyl-glucoside	51	0.24	0.16	0.35	0.05
Peonidin-3-*p*-coumaroyl-glucoside	51	0.18	0.16	0.21	0.01
Malvidin-3-*p*-coumaroyl-glucoside	51	0.49	0.18	0.77	0.16

**Table 4:** Number of latent variables, calibration, and validation results for the PLSR models obtained from processed spectra. RMSE values expressed as mg/g except for sugars, expressed as °Brix. Compounds in bold had R^2^_val_ greater than 0.70.

Compounds	#LV	R^2^_cal_	RMSE_cal_	R^2^_val_	RMSE_val_
**Must**					
Sugars	11	0.92	1.2	0.91	1.4
**Skins**					
Total phenols	4	0.76	1.7	0.73	1.8
Total flavonols	6	0.52	0.2	0.51	0.2
Delphinidin-3-glucoside	7	0.72	0.06	0.70	0.06
Cyanidin-3-glucoside	7	0.56	0.01	0.54	0.006
Petunidin-3-glucoside	9	0.75	0.08	0.73	0.09
Peonidin-3-glucoside	10	0.51	0.05	0.50	0.05
Malvidin-3-glucoside	6	0.75	0.3	0.72	0.3
Petunidin-3-*p*-coumaroyl-glucoside	5	0.78	0.02	0.76	0.02
*p*-Coumaric acid	7	0.67	0.004	0.65	0.004
Gallic acid	5	0.34	0.04	0.36	0.04
(+)-Catechin	7	0.63	0.01	0.62	0.01
**Seeds**					
Total phenols	12	0.82	1.8	0.83	1.7
Total flavanols	2	0.72	0.7	0.59	0.8
Gallic acid	4	0.63	0.02	0.70	0.02
(+)-Catechin	22	0.87	0.1	0.83	0.1
(−)-Epicatechin	2	0.69	0.2	0.69	0.2

## Data Availability

Not applicable.
